# The Broad Concept of “Spasticity-Plus Syndrome” in Multiple Sclerosis: A Possible New Concept in the Management of Multiple Sclerosis Symptoms

**DOI:** 10.3389/fneur.2020.00152

**Published:** 2020-03-17

**Authors:** Óscar Fernández, Lucienne Costa-Frossard, Marisa Martínez-Ginés, Paloma Montero, José Maria Prieto, Lluis Ramió

**Affiliations:** ^1^Biomedical Research Institute of Malaga, University of Málaga, Málaga, Spain; ^2^Department of Neurology, Ramón y Cajal University Hospital, Madrid, Spain; ^3^Department of Neurology, Gregorio Marañón Hospital, Madrid, Spain; ^4^Servicio de Neurología, Hospital Clínico San Carlos, Madrid, Spain; ^5^Servicio de Neurologia, Complejo Hospitalario Universitario de Santiago, Santiago de Compostela, Spain; ^6^Servicio de Neurologia, Hospital Universitari de Girona Doctor Josep Trueta, Girona, Spain

**Keywords:** multiple sclerosis, spasticity, symptomatic therapy, symptom cluster, symptomatic treatment

## Abstract

Multiple sclerosis (MS) pathology progressively affects multiple central nervous system (CNS) areas. Due to this fact, MS produces a wide array of symptoms. Symptomatic therapy of one MS symptom can cause or worsen other unwanted symptoms (anticholinergics used for bladder dysfunction produce impairment of cognition, many MS drugs produce erectile dysfunction, etc.). Appropriate symptomatic therapy is an unmet need. Several important functions/symptoms (muscle tone, sleep, bladder, pain) are mediated, in great part, in the brainstem. Cannabinoid receptors are distributed throughout the CNS irregularly: There is an accumulation of CB_1_ and CB_2_ receptors in the brainstem. Nabiximols (a combination of THC and CBD oromucosal spray) interact with both CB_1_ and CB_2_ receptors. In several clinical trials with Nabiximols for MS spasticity, the investigators report improvement not only in spasticity itself, but also in several functions/symptoms mentioned before (spasms, cramps, pain, gait, sleep, bladder function, fatigue, and possibly tremor). We can conceptualize and, therefore, hypothesize, through this indirect information, that it could be considered the existence of a broad “Spasticity-Plus Syndrome” that involves, a cluster of symptoms apart from spasticity itself, the rest of the mentioned functions/symptoms, probably because they are interlinked after the increase of muscle tone and mediated, at least in part, in the same or close areas of the brainstem. If this holds true, there exists the possibility to treat several spasticity-related symptoms induced by MS pathology with a single therapy, which would permit to avoid the unnecessary adverse effects produced by polytherapy. This would result in an important advance in the symptomatic management of MS.

In the last two decades, the availability of new disease-modifying therapies has radically changed the management of multiple sclerosis (MS) and relapsing–remitting MS in particular (1), resulting in a longer life expectancy for patients with the disease (2). Nevertheless, MS currently remains incurable and, in most patients, disability will eventually progress and they must live with the very many symptoms associated with the disease. These symptoms can have a major impact on patient's quality of life (3) and their management is considered important, although traditionally, this area has received far less attention than disease-modifying therapies (4).

A wide range of treatments are available to manage each of the MS symptoms (5–7). Given that different agents are used for different symptoms and a patient may have several symptoms present at the same time, many MS patients are multi-medicated, particularly as most patients will also be receiving disease-modifying therapies. This article will assess the current fragmented approaches to pharmacological management of spasticity muscle tone increase-related symptoms and their shortcomings. Given that the treatment of MS-associated muscle spasticity has been associated in a good number of clinical trials and also observational studies with the improvement of several other functions/symptoms present in MS (8), we will conceptualize, and subsequently hypothesize, about the clinical interest of introducing the more broad concept of “Spasticity-Plus Syndrome” to provide a unified framework for managing all these seemingly related functions/symptoms. By applying such a concept, it would be possible to simplify the management of symptoms associated with MS and reduce importantly the interactions and adverse effects associated with poly-medication.

## MS Symptoms

**Figure 1:**
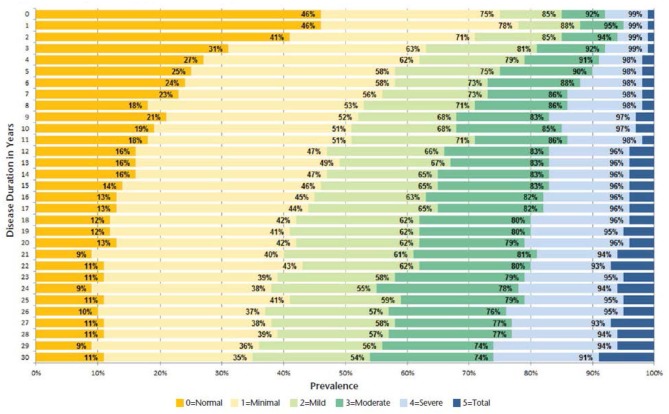
Spasticity prevalence according to disease duration in years (*N* = 23,842). With permission (9).

MS pathology affects multiple areas of the central nervous system (CNS), producing therefore a multiplicity of symptoms that can be basically classified as sensory alterations, fatigue, importantly cognitive dysfunction, pain (both paroxysmal and persistent), visual and brainstem symptoms (diplopia, oscillopsia, facial sensory symptoms, vertigo, and dizziness, nausea and vomiting, instability, etc.), those relating to mobility (spasticity, weakness, ataxia and tremor, impaired ambulation, and hand function), psychologic/psychiatric alterations (anxiety, depression, etc.), bowel, sexual and bladder dysfunctions, sleep disorders, and paroxysmal symptoms (seizures, dysarthria, etc.). All these symptoms vary along the course of the disease, being more prevalent as the disease evolves (Table 1) (9–12) (Figure 1).

**Table 1:** The percentage of symptoms present in multiple sclerosis vary along the course of the disease [adapted from (9–12)].

**Symptom**	**% (At onset-advanced)**
Sensory alterations	85–94
Fatigue	79–96
Cognitive dysfunction	63–81
Pain	57–85
Visual and brainstem symptoms (scotoma, diplopia, oscillopsia, vertigo, dizziness, etc.)	55–92
Motor alterations: spasticity, ataxia, tremor, impaired ambulation	50–91
Psychologic/psychiatric alterations (anxiety, depression, etc.)	50–79
Bowel alterations	41–82
Sexual dysfunction	40–90
Urinary dysfunction	40–87
Sleep disorders	40–60
Paroxysmal symptoms (seizures, dysarthria, etc.)	30–81

**Figure 2:**
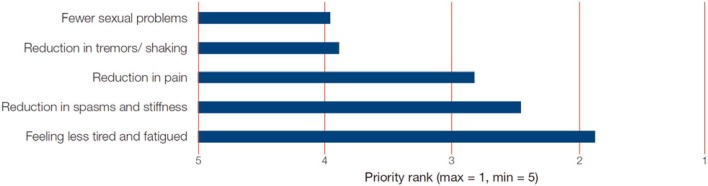
Symptom reduction priorities for patients. With permission (18).

Spasticity, a motor disorder characterized by a velocity-dependent increase in tonic stretch reflexes, due to the interruption of craniocaudal pathways by MS lesions at different CNS levels, is a very frequent symptom in patients with this disease. A large survey in the United States found 84% of patients with MS with some form of spasticity, with severity ranging from minimal (31%) to total (4%) (12). Similar results were obtained in a recent survey in the United Kingdom, which reported some form of spasticity in 86% of patients with MS (13). Another survey from the United Kingdom found that 47% of randomly selected patients with MS had clinically significant spasticity, defined as modified Ashworth scale score of 2, 3, or 4 (14). Spasticity is an important symptom of MS because it has a negative effect on mobility and can be painful (15, 16), which in turn is ranked highly as a concern among patients with MS and is considered to have a large impact on quality of life (3, 17, 18). Moreover, the muscle rigidity and spasms of spasticity trigger, worsen, or are associated to other functions/symptoms in MS subjects beyond mobility impairment, such as fatigue (3), sleep disorders, and bladder dysfunction (19) (Figure 2).

Ataxia, reported in up to 80% of MS patients at some point in their disease (20), and tremor, detected in more than a half of subjects in a sample of randomly selected patients with MS in a British study (21), are also impairing symptoms that can impact mobility.

Bladder symptoms have been reported in approximately three-quarters of patients with MS (22), while sexual dysfunction was found to be present in 84% of men and 85% of women (23). Both types of dysfunction can have a marked impact on quality of life, including among patients with otherwise low disability (24). Bowel dysfunction (including both constipation and fecal incontinence) was reported in 68% of an unselected patient population with MS (25).

Fatigue, depression, and cognitive impairment are also highly prevalent among patients with MS and impact quality of life even after accounting for physical disability (26, 27). Central pain in MS can be as severe as that associated with arthritic conditions (28) and the need for treatment may be underestimated.

In summary, symptoms of MS are widespread, varied, often interlinked, and highly prevalent among patients with the disease. The impact on quality of life is substantial, and mobility is a concern for patients (16, 17). MS symptoms have been cited as a major barrier for employment (29). Appropriate management of these symptoms is therefore imperative.

## Management of Spasticity Symptoms

As detailed above, the symptoms of MS are varied and substantially impact patients' well-being. Management of symptoms is, however, a complex task requiring a multidisciplinary approach. In some cases, non-pharmacological interventions may be beneficial, e.g., physiotherapy for spasticity, but might have a limited time effect and the evidence supporting such approaches is not always strong (30), and pharmacological interventions are often considered necessary. As shown in Table 2, a wide variety of agents can be used (31, 32).

**Table 2:** Commonly used pharmacological treatments for MS symptoms [adapted from (31, 32)].

**Symptom**	**Pharmacological treatment**
**MOBILITY-RELATED SYMPTOMS**	
Spasticity	Baclofen, tizanidine, nabiximols (THC:CBD), benzodiazepines (diazepam, clonazepam), gabapentin, dantrolene, botulinum toxin A (local treatment), intrathecal baclofen
Ataxia and tremor	Propranolol, clonazepam, levetiracetam, isoniazid, carbamazepine, ondansetron, dolasetron, cannabinoids, glutethimide
Impaired ambulation	Aminopyridines (fampridine)
**BLADDER, BOWEL, AND SEXUAL DYSFUNCTION**	
Urinary dysfunction	*Bladder inefficiency:* α1-blockers (indoramin) *Bladder overactivity:* antimuscarinics, intravesical botulinum toxin A, desmopressin, cannabinoids, intravesical vanilloids
Bowel dysfunction	Bulking agents, Laxatives
Sexual dysfunction	Sildenafil, tadalafil, vardenafil
**FATIGUE, COGNITIVE IMPAIRMENT, AND MOOD DISTURBANCE**	
Fatigue	Amantadine, modafinil, pemoline, aminopyridine, carnitine
Cognitive dysfunction	Acetylcholinesterase inhibitors, memantine, amantadine, pemoline, gingko biloba, L-amphetamine sulfate
Mood disturbance	Fluoxetine, sertraline, moclobemide
**PAIN**	
Paroxysmal pain	Carbamazepine, oxcarbazepine, lamotrigine, gabapentin, topiramate, misoprostol
Persistent pain	Amitriptyline, pregabalin, gabapentin, lamotrigine, levetiracetam, cannabinoids
**VISUAL AND BRAINSTEM SYMPTOMS**	
Visual dysfunction	Memantine, gabapentin
Brainstem-related symptoms	Antiepileptic drugs
**SLEEP DISORDERS**	
Excessive sleepiness Restless legs syndrome	Modafinil Dopaminergic agonists

Any pharmacological intervention has a risk of side effects (Table 3) and this risk is accentuated by drug–drug interaction possibilities. In some cases, the side effects from a drug to treat one MS symptom may exacerbate another symptom produced by the disease. For example, a number of treatments used for spasticity, fatigue, pain, and depression can all cause erectile dysfunction and decreased libido (Table 4) (33). An approach that would simplify the management of the diverse symptoms of MS-associated spasticity could potentially be beneficial for the patient.

**Table 3:** Main side effects of commonly used treatments for spasticity according to the EU Summary of Product Characteristics.

Baclofen	**Side effects**: depression, fatigue, ataxia, and tremor **Warnings**: Psychotic disorders, schizophrenia, depressive or manic disorders, confusional states or Parkinson's disease may be exacerbated by treatment **Drug–drug interactions**: muscle relaxants (such as tizanidine), with synthetic opiates or with alcohol, tricyclic antidepressants, anti-hypertensives
Tizanidine	**Side effects**: somnolence, dizziness, fatigue **Drug-drug interactions**: CYP inhibitors, antihypertensives, oral contraceptives
**Nabiximols (THC**:CBD)	**Side effects**: application site reactions, dizziness, fatigue, anxiety, disorientation, rare possibility of falls **Drug–drug interactions**: CYP inhibitor (ketoconazole, fluconazole, rifampicin, carbamazepine, phenytoin, phenobarbital, St John's Wort), alcohol, contraceptives
Diazepam	**Side effects**: Confusion, drowsiness, ataxia, impaired motor ability, tremor, fatigue, withdrawal symptoms **Drug–drug interactions**: alcohol, neuroleptics, anxiolytics/sedatives, hypnotics, antidepressants, anticonvulsants, sedating antihistamines, antipsychotics, baclofen tizanidine
Clonazepam	**Side effects**: Impaired concentration, restlessness, confusional state and disorientation, somnolence, slowed reaction, muscular hypotonia, dizziness, ataxia, light-headedness, co-ordination disturbances, fatigue and muscle weakness **Drug–drug interactions**: wide range of potential interactions with different classes of drug
Gabapentin	**Side effects**: confusion and emotional lability, depression, anxiety, nervousness, somnolence, dizziness, ataxia, convulsions, hyperkinesia, dysarthria, amnesia, tremor, insomnia, headache, coordination abnormal, nystagmus, increased, decreased, or absent reflexes, visual disturbances, diplopia, vertigo, arthralgia, myalgia, back pain, twitching, impotence, fatigue **Drug–drug interactions**: Opioids
Carbamazepine	**Side effects**: Ataxia, dizziness, somnolence; diplopia, headache; fatigue **Drug–drug interactions**: inhibitors or inducers of CYP 3A4
Levetiracetam	**Side effects**: Depression, hostility/aggression, anxiety, insomnia, nervousness/irritability, somnolence, headache, convulsion, balance disorder, dizziness, lethargy, tremor, asthenia/fatigue **Drug–drug interactions**: anti-epileptic medications
Dantrolene	**Side effects**: depression, confusion, insomnia, nervousness; seizure, visual disturbances, speech disturbances, headache **Drug–drug interactions**: non-depolarizing muscle relaxants
Botulinum toxin A	**Side effects**: Somnolence, gait disturbance, paresthesia (dependent on site of administration) **Drug–drug interactions**: Potentially with agents with neuromuscular blocking effects

**Table 4:** Adverse sexual function effects of drugs used in the symptomatic treatment of MS [adapted from (33)].

**Symptoms of MS**	**Treatment**	**Adverse sexual function effects associated with treatment**
Cognitive dysfunction	Donepezil	N/A
Spasticity	Baclofen Tizanidine Dantrolene Clonidine Benzodiazepines	ED, inability to ejaculate (rare) Urinary frequency, urgency, incontinence, urinary retention Decreased libido, ED, retrograde ejaculation N/A N/A
Fatigue	Amantadine Modafinil Methylphenidate Amphetamine/dextroamphetamine	Decreased libido N/A N/A ED, changes in libido (dose dependent)
Pain	Tricyclic antidepressants Valproic acid Carbamazepine Oxcarbazepine Lamotrigine Gabapentin Duloxetine	ED, ejaculatory impairment, anorgasmia, decreased libido ED ED N/A ED N/A Decreased libido, ED, ejaculation dysfunction and anorgasmia
Bladder and bowel dysfunction	Anticholinergic medication	Dry mouth, vaginal dryness, constipation
Depression	SSRIs Bupropion Venlafaxine	Decreased libido, anorgasmia, delayed ejaculation N/A ED, anorgasmia

*ED, erectile dysfunction; MS, multiple sclerosis; N/A, not applicable; SSRIs, selective serotonin reuptake inhibitors*.

## The Broad Concept of “Spasticity-Plus Syndrome” in MS

**Figure 3:**
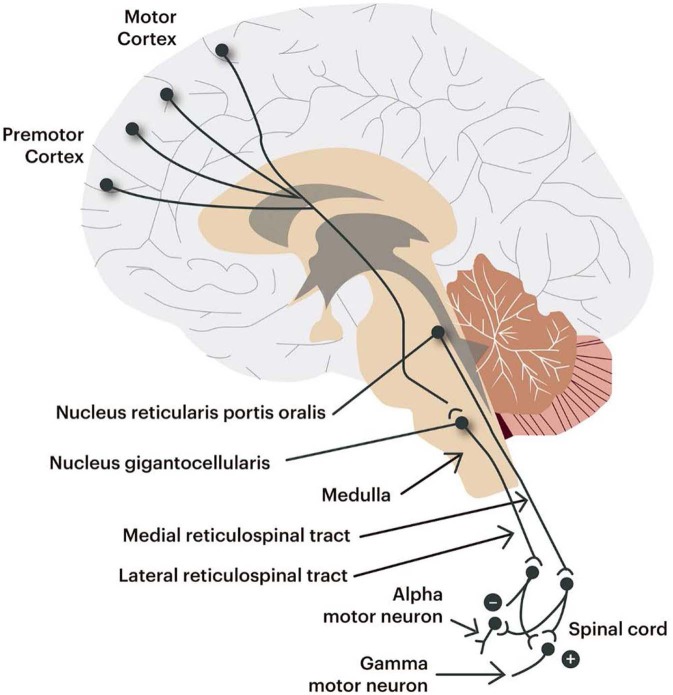
Areas of the CNS mediating spasticity.

A syndrome in medicine is classically defined as a combination of signs and/or symptoms that forms a distinct clinical picture indicative of a particular disease or disorder (34). Usually, these signs and/or symptoms would be considered to have a common underlying pathophysiology, or respond to a given therapy, although the clinical manifestations could be varied. In MS, spasticity is thought ultimately to arise from damage to motor areas or pathways, at multiple possible levels, in the CNS, leading to dynamic changes in motor circuit function and muscle tone that affect neuronal circuits and thereby cause spasticity (35). Bladder dysfunction in MS is originally caused by damaged neural pathways between the pons and sacral spinal cord, in turn impairing bladder function (36). Likewise, progressive demyelination and axonal or neuronal damage of the CNS leads to fatigue (37) and cognitive impairment (28). The presence of spasticity has been associated with worsening of other functions/symptoms in an epidemiological study of spasticity in MS (spasms, pain, bladder dysfunction, and sleep alterations) (3, 19) (Figure 3).

Although the specific mechanisms of the above symptoms might vary, damage to the CNS is common to all of them and we could consider, for practical reasons, as forming part of a syndrome, in which we would denote this new broad “Spasticity-Plus Syndrome” as a useful concept to be used in the symptomatic management in MS. This could be considered as an extension of the definition of symptom clusters or complexes, which underscores two primary features that have been defined as the existence of “three or more symptoms (e.g., pain, fatigue, sleep insufficiency) that are related to each other and that the symptoms must be inter-related through a common etiology or statistically as a cluster or latent variable. Such concurrent symptoms likely have a synergistic influence on behavioral, functional, and QOL outcomes and co-occurring symptoms seemingly provide a more efficient target for management than a single, isolated symptom taken out of its clinical context” (38).

The framing of MS symptoms within a syndrome also implies that a single intervention, in this case one that targets the cannabinoid system, a widely distributed molecular system in the CNS, may potentially influence a range of different symptoms. The presence in MS patients of one or more of the symptoms contained in the broad “Spasticity-Plus Syndrome” concept (spasticity and/or spasms-cramps and/or pain and/or bladder dysfunction and/or sleep disorders and/or fatigue and/or tremor) would have to trigger in physicians the search of the other symptoms' presence and severity and the attempt to manage them as appropriately as possible with proven, well-tolerated, and as simple as possible to use options.

**Figure 4:**
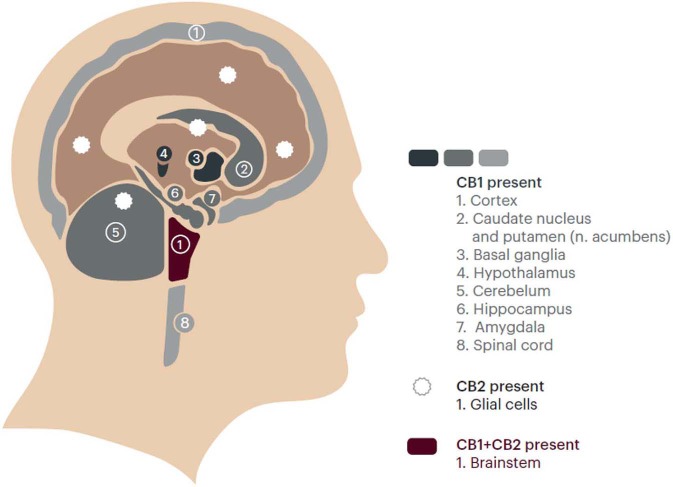
Distribution of cannabinoid receptors (CB1 and CB2) in the central nervous system (The Endocannabinoid System). Redrawn from https://www.fundacion-canna.es/en/endocannabinoid-system. Accessed April 06, 2019). With permission.

The cannabinoid system is present in the brain, spinal cord, and peripheral nerves and comprises the cannabinoid receptors, CB_1_ and CB_2_, along with their ligands, the endocannabinoids, which are derived from fatty acids (39, 40). CB_1_ receptors are widely distributed within the CNS on nerve terminals, including areas associated with movement, postural control, pain and sensory perception, memory, cognition, emotion, appetite, and autonomic and endocrine function. A particularly high accumulation of CB_1_ and CB_2_ receptors is found in the brainstem where important functions/symptoms such as spasticity, sleep, bladder, and pain are mediated (41). CB_2_ receptors are mainly involved in regulating cytokine release from immune cells and immune cell migration in a manner that appears to reduce inflammation and certain kinds of pain (42). In short, the possible new and broad concept of “Spasticity-Plus Syndrome” in MS may point us toward an approach for simplifying management of MS symptoms (Figure 4).

## Cannabinoids for the Treatment of Functions/Symptoms Belonging to the Broad “Spasticity-Plus Syndrome”

Several randomized clinical trials have demonstrated improvement in resistant spasticity symptoms, in patients with MS following add-on treatment with an oromucosal spray containing a 1:1 mixture of 9-δ-tetrahydocannabinol and cannabidiol (THC:CBD) (43–47).

This benefit for spasticity has also been reported in very many observational trials in the clinical practice setting (48). Studies of THC:CBD for the treatment of MS symptoms, other than spasticity itself, are fewer and often beset with design limitations, such as small number of patients. Nevertheless, large THC:CBD studies that collected evolution of pain, sleep disorders, and bladder dysfunction as secondary endpoints do point to a potential benefit for these symptoms (43–46). A substudy of the ***CA**nnabinoids for treatment of spasticity and other symptoms related to* ***M**ultiple* ***S**clerosis* study (“CAMS”) (49) found significant benefit in the control of incontinence, although THC-only tolerability profile is less interesting than the THC and CBD combination (50, 51). In the case of MS-associated pain, a systematic review of randomized clinical trials of cannabinoid treatments concluded that these agents are effective in alleviating pain (52). There is thus evidence that THC:CBD can be used to treat a variety of MS spasticity-associated and somehow related symptoms (53). Moreover, application of the possible new broad concept of “Spasticity-Plus Syndrome” in MS would suggest an appropriate line of investigation, in patients with more than one symptom that could be amenable to a single therapy, to simplify symptom management.

As a clear limitation, we consider this as a preliminary conceptual proposal that has to be sustained in the future with new studies, not yet available, and that could give more background and support to our concept, so that the hypothesis would be testable and be a promising area of research in the field of symptomatic therapy. Another limitation is the fact that we do not know whether this concept could be applied to the spasticity present in other diseases such as spinal cord injury, stroke, etc., as it has not been surveyed yet as far as we know.

## Conclusions

The numerous and varied symptoms associated with MS requires complex management with multiple drugs, all with potential side effects that may exacerbate other symptoms and with potential drug–drug interactions. Recognition that a good number of MS symptoms might have a common or close underlying pathophysiology, or respond to a single therapy, in the form of a new broad “Spastic-Plus Syndrome” in MS may help simplify treatment of these symptoms with agents such as cannabinoids that target CB_1_ and CB_2_ receptors.

## Author Contributions

ÓF, LC-F, MM-G, PM, JP, and LR contributed conception and design of the study. ÓF wrote the first draft of the manuscript. All authors contributed to manuscript revision, read, and approved the submitted version.

### Conflict of Interest

ÓF has received honoraria as consultant in advisory boards, as chair/lecturer in meetings, from participation in clinical trials and other research projects promoted by Actelion, Allergan, Almirall, Bayer-Schering, Biogen, Merck Serono, Novartis, Sanofi Genzyme, Roche, Teva, Orizon, and Araclon, and research support from the Hospital Foundation FIMABIS. LC-F has received compensation for consulting services and speaking fees from Merck, Novartis, Biogen, Bayer, Sanofi, Genzyme, TEVA, Almirall, Biopas, Ipsen, Celgene, and Mylan. MM-G has received compensation for consulting services and speaking fees from Merck, Biogen, Novartis, Sanofi-Genzyme, Almirall, Roche, and Teva. PM has received compensation for consulting services and speaking fees from Almirall. JP has done consultancy work for Bayer HealthCare, Biogen, Genzyme, Novartis, Sanofi-Aventis, Teva, Roche, Merck, and Almirall; has given lectures in congresses and symposia organized by Almirall, Bayer, Biogen, Genzyme, Merck, Novartis, Sanofi-Aventis, and Teva Pharmaceuticals; and has received funding for research projects from Almirall, Biogen, Novartis, and Sanofi-Genzyme. LR has received compensation for consulting services and speaking fees from Biogen, Novartis, Bayer, Merck, Sanofi, Genzyme, TEVA, Almirall, and Mylan.

## References

[1] Ransohoff RM Hafler DA Lucchinetti CF Multiple sclerosis-a quiet revolution. Nat Rev Neurol. (2015) 11:134–42. 10.1038/nrneurol.2015.14 25686758 PMC4556342

[2] Lunde HMB Assmus J Myhr K-M Bø L Grytten N. Survival and cause of death in multiple sclerosis: a 60-year longitudinal population study. J Neurol Neurosurg Psychiatr. (2017) 88:621–5. 10.1136/jnnp-2016-315238 28365589 PMC5537547

[3] Flachenecker P Henze T Zettl UK. Spasticity in patients with multiple sclerosis–clinical characteristics, treatment and quality of life. Acta Neurol Scand. (2014) 129:154–62. 10.1111/ane.12202 24256407

[4] Fox RJ Thompson A Baker D Baneke P Brown D Browne P. Setting a research agenda for progressive multiple sclerosis: the international collaborative on progressive MS. Mult Scler. (2012) 18:1534–40. 10.1177/1352458512458169 22917690 PMC3573679

[5] Ben-Zacharia AB. Therapeutics for multiple sclerosis symptoms. Mt Sinai J Med. (2011) 78:176–91. 10.1002/msj.20245 21425263

[6] Thompson AJ Toosy AT Ciccarelli O. Pharmacological management of symptoms in multiple sclerosis: current approaches and future directions. Lancet Neurol. (2010) 9:1182–99. 10.1016/S1474-4422(10)70249-0 21087742

[7] Toosy A Ciccarelli O Thompson A Symptomatic treatment and management of multiple sclerosis. In: Goodin DS, editor. Handbook of Clinical Neurology Multiple Sclerosis Related Disorders, 3rd series, Vol. 122 Amsterdam: Elsevier B.V (2014). p. 514–61. 10.1016/B978-0-444-52001-2.00023-6 24507534

[8] Marková J. Newest evidence for tetrahydrocannabinol:cannabidiol oromucosal spray from randomized clinical trials. Neurodegener Dis Manag. (2019) 9:9–13. 10.2217/nmt-2018-0050 30657024

[9] Kister I Bacon TE Chamot E Salter AR Cutter GR Kalina JT. Natural history of multiple sclerosis symptoms. Int J MS Care. (2013) 15:146–56. 10.7224/1537-2073.2012-053 24453777 PMC3883021

[10] Sastre-Garriga J Tintoré M Nos C Tur C Río J Téllez N. Clinical features of CIS of the brainstem/cerebellum of the kind seen in MS. J Neurol. (2010) 257:742–6. 10.1007/s00415-009-5403-0 19946780

[11] Nociti V Losavio FA Gnoni V Losurdo A Testani E Vollono C. Sleep and fatigue in multiple sclerosis: a questionnaire-based, cross-sectional, cohort study. J Neurol Sci. (2017) 372:387–92. 10.1016/j.jns.2016.10.040 27823835

[12] Rizzo MA Hadjimichael OC Preiningerova J Vollmer TL. Prevalence and treatment of spasticity reported by multiple sclerosis patients. Mult Scler Houndmills Basingstoke Engl. (2004) 10:589–95. 10.1191/1352458504ms1085oa 15471378

[13] Milinis K Tennant A Young CA TONiC study group. spasticity in multiple sclerosis: associations with impairments and overall quality of life. Mult Scler Relat Disord. (2016). 5:34–9. 10.1016/j.msard.2015.10.007 26856941

[14] Barnes MP Kent RM Semlyen JK McMullen KM. Spasticity in multiple sclerosis. Neurorehabil Neural Repair. (2003) 17:66–70. 10.1177/0888439002250449 12645447

[15] Sosnoff JJ Gappmaier E Frame A Motl RW. Influence of spasticity on mobility and balance in persons with multiple sclerosis. J Neurol Phys Ther JNPT. (2011) 35:129–32. 10.1097/NPT.0b013e31822a8c40 21934374

[16] Zwibel HL. Contribution of impaired mobility and general symptoms to the burden of multiple sclerosis. Adv Ther. (2009) 26:1043–57. 10.1007/s12325-009-0082-x 20082242

[17] Heesen C Böhm J Reich C Kasper J Goebel M Gold SM. Patient perception of bodily functions in multiple sclerosis: gait and visual function are the most valuable. Mult Scler Houndmills Basingstoke Engl. (2008) 14:988–91. 10.1177/1352458508088916 18505775

[18] Heller M Taylor D Greater Expectations: The Future Hopes of People With Multiple Sclerosis. (2017). Available online at: https://pdfs.semanticscholar.org/866a/959d88a224883f7e9dd5ddbec31017f36bac.pdf

[19] Oreja-Guevara C González-Segura D Vila C. Spasticity in multiple sclerosis: results of a patient survey. Int J Neurosci. (2013) 123:400–8. 10.3109/00207454.2012.762364 23297730

[20] Mills RJ Yap L Young CA Treatment for ataxia in multiple sclerosis. Cochrane Database Syst Rev. (2007) 24:CD005029 10.1002/14651858.CD005029.pub2 17253537

[21] Alusi SH Worthington J Glickman S Bain PG A study of tremor in multiple sclerosis. Brain J Neurol. (2001) 124:720–30. 10.1093/brain/124.4.720 11287372

[22] DasGupta R Fowler CJ. Bladder, bowel and sexual dysfunction in multiple sclerosis: management strategies. Drugs. (2003) 63:153–66. 10.2165/00003495-200363020-00003 12515563

[23] Tepavcevic DK Kostic J Basuroski ID Stojsavljevic N Pekmezovic T Drulovic J. The impact of sexual dysfunction on the quality of life measured by MSQoL-54 in patients with multiple sclerosis. Mult Scler Houndmills Basingstoke Engl. (2008) 14:1131–6. 10.1177/1352458508093619 18632783

[24] Nortvedt MW Riise T Myhr KM Landtblom AM Bakke A Nyland HI. Reduced quality of life among multiple sclerosis patients with sexual disturbance and bladder dysfunction. Mult Scler Houndmills Basingstoke Engl. (2001) 7:231–5. 10.1177/135245850100700404 11548982

[25] Hinds JP Eidelman BH Wald A. Prevalence of bowel dysfunction in multiple sclerosis. a population survey. Gastroenterology. (1990) 98:1538–42. 10.1016/0016-5085(90)91087-M 2338192

[26] Bakshi R. Fatigue associated with multiple sclerosis: diagnosis, impact and management. Mult Scler. (2003) 9:219–27. 10.1191/1352458503ms904oa 12814166

[27] Chiaravalloti ND DeLuca J. Cognitive impairment in multiple sclerosis. Lancet Neurol. (2008) 7:1139–51. 10.1016/S1474-4422(08)70259-X 19007738

[28] Kalia LV O'Connor PW. Severity of chronic pain and its relationship to quality of life in multiple sclerosis. Mult Scler. (2005) 11:322–7. 10.1191/1352458505ms1168oa 15957515

[29] Simmons RD Tribe KL McDonald EA. Living with multiple sclerosis: longitudinal changes in employment and the importance of symptom management. J Neurol. (2010) 257:926–36. 10.1007/s00415-009-5441-7 20084515

[30] Amatya B Khan F Galea M. Rehabilitation for people with multiple sclerosis: an overview of cochrane reviews. Cochr Database of Syst Rev. (2019) 1:CD012732. 10.1002/14651858.CD012732.pub2 30637728 PMC6353175

[31] Newsome SD Aliotta PJ Bainbridge J Bennett SE Cutter G Fenton K. A framework of care in multiple sclerosis, part 2: symptomatic care and beyond. Int J MS Care. (2017) 19:42–56. 10.7224/1537-2073.2016-062 28243186 PMC5315323

[32] Oreja-Guevara C Montalban X de Andrés C Casanova-Estruch B Muñoz-García D García I. Consensus document on spasticity in patients with multiple sclerosis. grupo de enfermedades desmielinizantes de la sociedad española de neurología. Rev Neurol. (2013) 57:359–73. 10.33588/rn.5708.2013374 24081891

[33] Fletcher SG Castro-Borrero W Remington G Treadaway K Lemack GE Frohman EM. Sexual dysfunction in patients with multiple sclerosis: a multidisciplinary approach to evaluation and management. Nat Clin Pract Urol. (2009) 6:96–107. 10.1038/ncpuro1298 19198623

[34] The British Medical Association Illustrated Medical Dictionary. London: Dorling Kindersley (2002). p. 177–536.

[35] Patejdl R Zettl UK. Spasticity in multiple sclerosis: contribution of inflammation, autoimmune mediated neuronal damage and therapeutic interventions. Autoimmun Rev. (2017) 16:925–36. 10.1016/j.autrev.2017.07.004 28698092

[36] Betts CD D'Mellow MT Fowler CJ. Urinary symptoms and the neurological features of bladder dysfunction in multiple sclerosis. J Neurol Neurosurg Psychiatr. (1993) 56:245–50. 10.1136/jnnp.56.3.245 8459239 PMC1014855

[37] Patejdl R Penner IK Noack TK Zettl UK. Multiple sclerosis and fatigue: a review on the contribution of inflammation and immune-mediated neurodegeneration. Autoimmun Rev. (2016) 15:210–20. 10.1016/j.autrev.2015.11.005 26589194

[38] Motl RW Suh Y Weikert M. Symptom cluster and quality of life in multiple sclerosis. J Pain Symptom Manage. (2010) 39:1025–32. 10.1016/j.jpainsymman.2009.11.312 20434872

[39] Svízenská I Dubový P Sulcová A. Cannabinoid receptors 1 and 2 (CB1 and CB2), their distribution, ligands and functional involvement in nervous system structures–a short review. Pharmacol Biochem Behav. (2008) 90:501–11. 10.1016/j.pbb.2008.05.010 18584858

[40] Howlett AC. The cannabinoid receptors. Prostaglandins Other Lipid Mediat. (2002) 68–69:619–31. 10.1016/S0090-6980(02)00060-6 12432948

[41] van Sickle MD Duncan M Kingsley PJ Mouihate A Urbani P Mackie K. Identification and functional characterization of brainstem cannabinoid CB2 receptors. Science. (2005) 310:329–32. 10.1126/science.1115740 16224028

[42] Baker D Jackson SJ Pryce G. Cannabinoid control of neuroinflammation related to multiple sclerosis. Br J Pharmacol. (2007) 152:649–54. 10.1038/sj.bjp.0707458 17891167 PMC2190016

[43] Wade DT Makela P Robson P House H Bateman C. Do cannabis-based medicinal extracts have general or specific effects on symptoms inmultiple sclerosis? A double-blind, randomized, placebo-controlled study on 160 patients. Mult Scler. (2004) 10:434–41. 10.1191/1352458504ms1082oa 15327042

[44] Collin C Davies P Mutiboko IK Ratcliffe S Sativex spasticity in MS study group. randomized controlled trial of cannabis-based medicine in spasticity caused by multiple sclerosis. Eur J Neurol. (2007) 14:290–6. 10.1111/j.1468-1331.2006.01639.x 17355549

[45] Collin C Ehler E Waberzinek G Alsindi Z Davies P Powell K. A double-blind, randomized, placebo-controlled, parallel-group study of sativex, in subjects with symptoms of spasticity due to multiple sclerosis. Neurol Res. (2010) 32:451–9. 10.1179/016164109X12590518685660 20307378

[46] Novotna A Mares J Ratcliffe S Novakova I Vachova M Zapletalova O. A randomized, double-blind, placebo-controlled, parallel-group, enriched-design study of nabiximols^*^ (Sativex(®)), as add-on therapy, in subjects with refractory spasticity caused by multiple sclerosis. Eur J Neurol. (2011) 18:1122–31. 10.1111/j.1468-1331.2010.03328.x 21362108

[47] Markovà J Essner U Akmaz B Marinelli M Trompke C Lentschat A. Sativex® as add-on therapy vs. further optimized first-line ANTispastics (SAVANT) in resistant multiple sclerosis spasticity: a double-blind, placebo-controlled randomised clinical trial. Int J Neurosci. (2019) 129:119–28. 10.1080/00207454.2018.1481066 29792372

[48] Fernández O. Advances in the management of MS spasticity: recent observational studies. Eur Neurol. (2014) 72(Suppl. 1):12–4. 10.1159/000367618 25278118

[49] Zajicek J Fox P Sanders H Wright D Vickery J Nunn A. Cannabinoids for treatment of spasticity and other symptoms related to multiple sclerosis (CAMS study): multicentre randomised placebo-controlled trial. Lancet. (2003) 362:1517–26. 10.1016/S0140-6736(03)14738-1 14615106

[50] Freeman RM Adekanmi O Waterfield MR Waterfield AE Wright D Zajicek J. The effect of cannabis on urge incontinence in patients with multiple sclerosis: a multicentre, randomised placebo-controlled trial (CAMS-LUTS). Int Urogynecol J Pelvic Floor Dysfunct. (2006) 17:636–41. 10.1007/s00192-006-0086-x 16552618

[51] Kavia RB De Ridder D Constantinescu CS Stott CG Fowler CJ. Randomized controlled trial of sativex to treat detrusor overactivity in multiple sclerosis. Mult Scler. (2010) 16:1349–59. 10.1177/1352458510378020 20829244

[52] Iskedjian M Bereza B Gordon A Piwko C Einarson TR. Meta-analysis of cannabis based treatments for neuropathic and multiple sclerosis-related pain. Curr Med Res Opin. (2007) 23:17–24. 10.1185/030079906X158066 17257464

[53] Arroyo R Vila C Dechant KL. Impact of Sativex(®) on quality of life and activities of daily living in patients with multiple sclerosis spasticity. J Comp Eff Res. (2014) 3:435–44. 10.2217/cer.14.30 25275238

